# Change in spring arrival of migratory birds under an era of climate change, Swedish data from the last 140 years

**DOI:** 10.1007/s13280-014-0600-1

**Published:** 2015-01-09

**Authors:** Cecilia Kullberg, Thord Fransson, Johanna Hedlund, Niclas Jonzén, Ola Langvall, Johan Nilsson, Kjell Bolmgren

**Affiliations:** 1 Department of Zoology, Stockholm University, 106 91 Stockholm, Sweden; 2 Department of Environmental Research and Monitoring, Swedish Museum of Natural History, Stockholm, Sweden; 3 Theoretical Population Ecology and Evolution Group, Department of Biology, Lund University, Lund, Sweden; 4 Unit for Field-Based Forest Research, Swedish University of Agricultural Sciences, Uppsala, Sweden; 5 The Swedish Species Information Centre, Swedish University of Agricultural Sciences, Uppsala, Sweden; 6 Department of Ecology, Environment and Plant Sciences, Stockholm University, Stockholm, Sweden

**Keywords:** Bird migration, Phenology, Migratory strategy, Climate changes, Spring arrival

## Abstract

Many migratory bird species have advanced their spring arrival during the latest decades, most probably due to climate change. However, studies on migratory phenology in the period before recent global warming are scarce. We have analyzed a historical dataset (1873–1917) of spring arrival to southern and central Sweden of 14 migratory bird species. In addition, we have used relative differences between historical and present-day observations (1984–2013) to evaluate the effect of latitude and migratory strategy on day of arrival over time. There was a larger change in spring phenology in short-distance migrants than in long-distance migrants. Interestingly, the results further suggest that climate change has affected the phenology of short-distance migrants more in southern than in central Sweden. The results suggest that the much earlier calculated arrival to southern Sweden among short-distance migrants mirrors a change in location of wintering areas, hence, connecting migration phenology and wintering range shifts.

## Introduction

There is ample evidence that climate change has caused phenological shifts that eventually can result in change in distribution or abundance of species (Walther et al. 2002; Parmesan 2006; Willis et al. 2008; Lehikoinen and Sparks 2010). In birds, and especially migrants, there is a large body of scientific evidence for the effect of climate change on phenology and population trends (Root et al. 2003; Parmesan and Yohe 2003; Möller et al. 2008; Both et al. 2010). In many areas, especially in Europe and North America, spring events like budburst and caterpillar availability occur earlier during the latest decades due to warmer temperatures (Visser et al. 1998; Bauer et al. 2010), and many migratory bird species are advancing their arrival to the breeding ground (Cotton 2003; Parmesan and Yohe 2003; Jonzén et al. 2006; Knudsen et al. 2011).

For a number of reasons, including competition for territories and fitness cost of early egg-laying (e.g., Jonzén et al. 2007; Johansson and Jonzén 2012; Visser et al. 2012), the advancement in bird arrival dates is sometimes not as large as the earlier occurrence of the food peak, but may still be an adaptive response to climate change. If the time between arrival and breeding is limited, the consequence may be a temporal mismatch between breeding time and food availability, leading to situations where chicks are reared under suboptimal conditions (Both and Visser 2001; Strode 2003; Both et al. 2006).

Several studies show that spring arrival in long-distance migrants is less affected by temperature than in short-distance migrants (Nott et al. 2002; Tryanowski et al. 2002; Hubalek 2004; Miller-Rushing et al. 2008b). This has been suggested to be a result of long-distance migrants being more restricted by their endogenous time program (Both and Visser 2001; Coppack and Both 2002; Jenni and Kéry 2003). Short-distance migrants have also been shown to display a more flexible behavioral response to local weather at stopover sites (Calvert et al. 2012). Larger ecological mismatches at the breeding grounds have thus been suggested to explain the observation of negative population trends in long-distance migrants (Both et al. 2010; Saino et al. 2011). However, it has also been shown that trans-Saharan migrants can advance spring arrival to the same extent as short-distance migrants in recent years, suggesting that onset of migration in long-distance migrants may be more flexible than previously assumed (Jonzén et al. 2006).

The vast majority of published studies on migratory phenology in birds concern the last fifty years, a period when global warming has escalated (Hansen et al. 2005; Rahmstorf 2008). Historical data on bird migration phenology before the middle of the 20th century, when recent global warming started, are scarce. There are, however, some studies on historical data revealing that migratory birds tended to arrive earlier in warmer springs also in the 18th and 19th centuries (Sparks 1999; Ellwood et al. 2010). Analyzing data from a variety of data sources and interpretation of volunteer and bird-club records must be performed with care. For example, many of these type of records are documentations of first arrival dates which may be affected by both population size of the species and sampling effort (Tryjanowski and Sparks 2001; Miller-Rushing et al. 2008a, c), and these factors as well as the information on how data were gathered will of course be even harder to track in historical datasets (Miller-Rushing et al. 2012). Probably due to these difficulties, many historical data are underutilized, although they represent a possibility to provide insights into climate change impacts, given that right methods are used (Primack and Miller-Rushing 2012).

Here we analyze a uniquely standardized dataset based on observations of spring arrival of 14 migratory bird species to Sweden 1873–1917. In order to evaluate changes in arrival date between historical and present-day datasets, we have in addition analyzed recent data on spring observations on the same species during the years 1984–2013. To evaluate if a phenological change differ depending on latitude (Parmesan 2007; Rubolini et al. 2007), we have specifically looked at the relative difference between arrival date between the historical and present-day data at different latitudes. Furthermore, we study if there are differences in latitudinal effect over time depending on migratory strategy by comparing data from both long- and short-distance migrants.

## Materials and methods

### Historical data

Historical records on first arrival dates for 14 migratory bird species (Table 1) were provided by the Swedish National Phenology Network (headed by the Swedish University of Agricultural Sciences, SLU). The material was collected by observers organized in a governmentally led national network using the same instructions and submission forms, mainly reporting not only phenology of plants, but also some common migratory birds on a yearly basis from 1865 to 1951. However, as the network was formally launched in 1873, we decided to exclude earlier observations. We have not found out when, or if, the network was ever formally ended, but the participation drops markedly after about 40 years. Lacking any other information we decided to use 1917 as the cut-off point, the same year used by Arnell (1923) in analyses of plant phenology. The phenological data on birds have never been published except for a general analysis of the data from 1873 to 1877 (Carlheim-Gyllenskiöld 1879) and information and summaries of the avian data set (Arnell 1877). To exclude potential low-quality observations we only included data from sites with observations that comprised 10 years or more. As data were heavily skewed toward the south of Sweden, we decided to exclude data from above 61°N, giving us data in southern to central Sweden ranging over 5.5° latitudes. After these reductions, the data on the 14 species comprised 15 773 bird arrival observations distributed over 103 sites.

**Table 1:** Included migratory species in the study with categorisation of migratory strategy (long-distance migrants pass the Saharan desert, while the short-distance migrants winter north of Sahara). Period of included observations/year in present-day dataset starts at January 1 and ends according to the dates in the table (SOF 2002). The table further includes intercept and regression coefficients for the effect of latitude on first arrival date for each species in the historical and present-day dataset, respectively. Values from the historical data model were obtained from a linear mixed model per species, and values from the present day model were derived as mean values from linear quantile regression models for each year and species

Species no.	Species	Migratory strategy	End of obs. dates	Historical dataset		Present-day dataset	
				Intercept	Regr. coeff.	Intercept	Regr. coeff.
1	Common Chaffinch (*Fringilla coelebs*)	Short	May 15	−78.0	2.80	−324.6	5.86
2	Stock Dove (*Columba oenas*)	Short	April 30	−102.7	3.20	−409.6	7.90
3	Common Starling (*Sturnus vulgaris*)	Short	April 30	−176.6	4.29	−463.0	8.62
4	Eurasian Skylark (*Alauda arvensis*)	Short	April 30	−219.1	4.86	−360.0	7.13
5	Whooper Swan (*Cygnus cygnus*)	Short	May 15	−265.7	6.09	−315.0	5.49
6	Eurasian Woodcock (*Scolopax rusticola*)	Short	May 31	−167.2	4.46	−601.3	10.88
7	White Wagtail (*Motacilla alba alba*)	Short	May 31	100.2	0.04	−37.1	2.18
8	Common Crane (*Grus grus*)	Short	April 30	−34.4	2.38	−83.5	2.83
9	Yellow Wagtail (*Motacilla flava)*	Long	May 31	33.2	1.54	63.4	0.91
10	Common Cuckoo (*Cuculus canorus*)	Long	May 31	91.7	0.66	77.5	0.82
11	Common House Martin (*Delichon urbicum*)	Long	May 31	58.6	1.27	49.2	1.12
12	Common Redstart (*Phoenicurus phoenicurus*)	Long	May 31	92.5	0.51	−7.9	2.08
13	Northern Wheatear (*Oenanthe oenanthe*)	Long	May 15	−54.3	2.83	0.9	1.70
14	Common Swift (*Apus apus*)	Long	June 15	151.0	−0.14	118.5	0.18

### Present-day data

Observational data on the same 14 bird species were obtained from a contemporary dataset from 1984 to 2013 provided by the Species Observation System, Swedish Species Information Centre. The Species Observation System is a web-based platform where the public can report sightings of Swedish plants, animals, and fungi (http://www.artportalen.se). As with the historical data, to obtain high-quality data from experienced and accurate observers, we only used observers with observation sites that had at least 10 years of data. Furthermore, since the data from the Swedish Observation System are based on sightings over the whole year, without any instruction to the observers to report first sightings per year, we have only used the first report per year and observation site. We omitted data on first reports after a specified cut-off date in spring, to omit obvious summer and autumn observations. This species-specific cut-off date was taken from data on the migratory period obtained from the Swedish Ornithological Society (SOF 2002; Table 1). Thus, only reports between the first of January and this specific date were used in the analyses. Just as in the evaluation of the historical dataset, data from above 61°N were removed from the analysis as observations were heavily skewed toward the south of Sweden. After these reductions, the data resulted in 85 086 observations from 5 299 sites for the 14 species under study.

### Temperature data

To illustrate climatic conditions in the two study periods, average temperature per month and year were obtained from the Swedish Meteorological and Hydrological Institute (SMHI). Data were obtained for the two study periods at three sites in central Sweden (Stockholm 59.3°N 18.1°E; Uppsala 59.9°N 17.6°E; Karlstad 59.4°N 13.5°E) and three sites in southern Sweden (Falsterbo 55.4°N 12.8°E; Halmstad 56.7°N 12.9°E; Kalmar 56.7°N 16.3°E).

### Statistical analyses

**Fig. 1:**
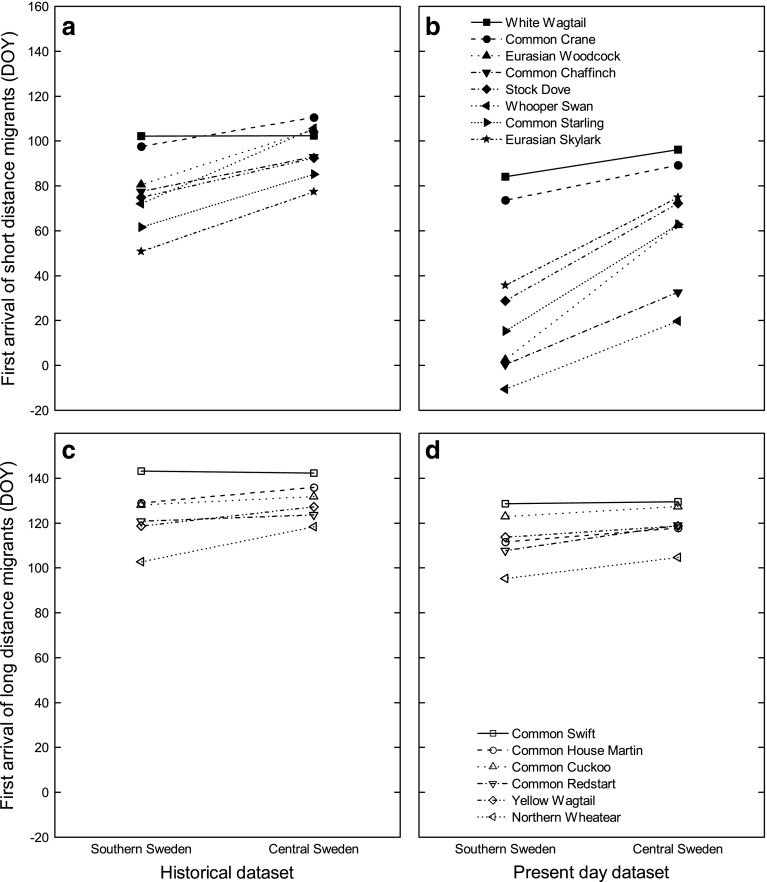
Regression lines for estimated first arrival day of year (DOY) in relation to latitude for the historical (**a**, **c**) and present day (**b**, **d**) datasets in short-distance (**a**, **b**) and long-distance (**c**, **d**) migrants, respectively. Southern Sweden = 55.5°N and central Sweden = 61°N. DOY 50 represents February 19, while DOY 100 represents April 10

For the historical data, we used a linear mixed model analysis (R Core Team 2014; Bates et al. 2014) examining the relation between arrival day and the following factors: latitude, altitude, and distance to larger water bodies. Factors were nested within year. Data on altitude and distance to large water bodies had a highly skewed distribution and were thus square-root transformed. Since present-day data were not based on instruction for first sightings, we used quantile regression (Koenker 2013) to analyze patterns of early reports in relation to latitude. With the linear quantile model, we obtained a regression for a given percentile of the dataset giving us an estimate of arrival date in relation to latitude for each species. We chose to use the 5% percentile of the data for this estimate. In accordance with the historical dataset, we used latitude, altitude (square-root transformed), and distance to larger water bodies (square-root transformed) as factors also for the present-day dataset. After obtaining intercept and regression coefficients for the effect of latitude on arrival date for each species and year, we calculated mean intercept and coefficient for each species in the present-day data. The obtained intercept and coefficient for each species from the historical and present-day datasets were then used to calculate arrival date at specific latitudes and analyzed to reveal patterns of change depending on migratory strategy of the species under study (Table 1; Fig. 1). Since the two datasets contain data that were gathered differently using different methods, we cannot compare the calculated arrival dates of the two models directly. We therefore present modeled arrival dates and perform statistical analyses only on relative differences between the historical and present day model in the results. When analyzing effects of migratory strategy, we compared trans-Saharan migrants (long-distance migrants; 6 species) with migrants that do not cross the desert (short-distance migrants; 8 species).

When analyzing climatic conditions in the two study periods, we have calculated and compared average temperature per month and year, based on data from three sites in southern and central Sweden, respectively.

## Results

**Fig. 2:**
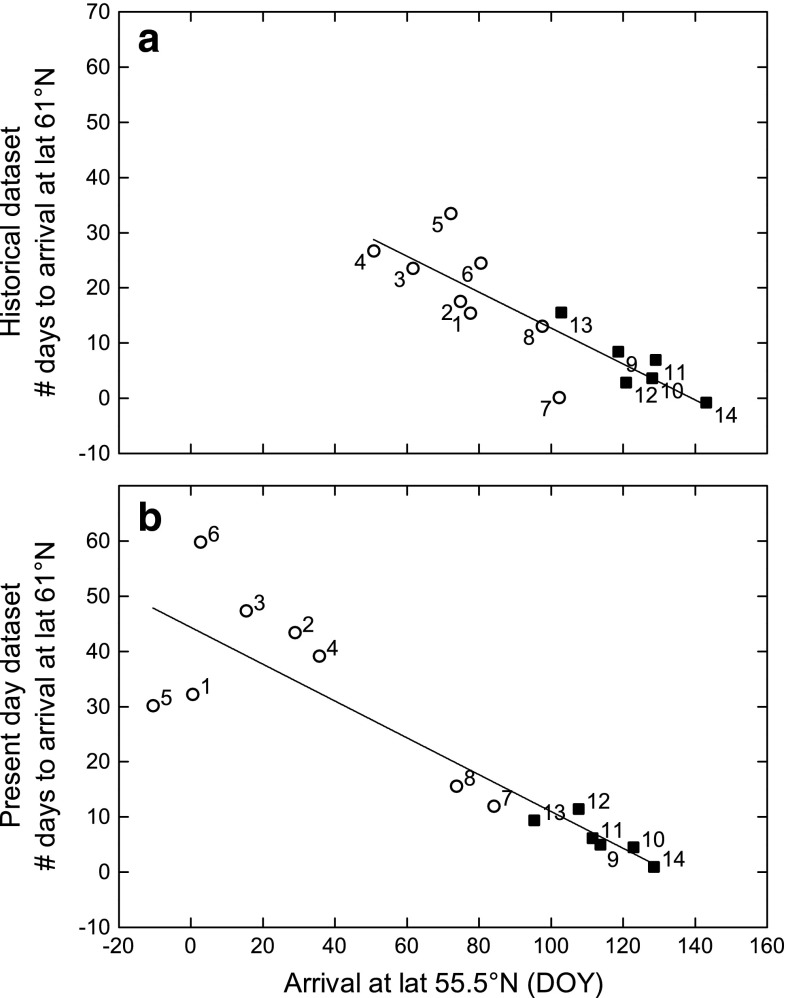
Difference in days between first arrival to southern and central Sweden in relation to first arrival day of year (DOY) in southern Sweden for each species, according to the historical (**a**) and present day (**b**) model. Numbers at each marker correspond to the species number in Table 1. *Open circles* represent short-distance migrants and *filled squares* represent long-distance migrants. DOY 50 represents February 19, while DOY 100 represents April 10

There was a strong negative relationship between first arrival date in southern Sweden and how many days later the species arrive to central Sweden, both in the historical model (linear regression; *n* = 14, *r* ^2^ = 0.74, *p* < 0.0001; Fig. 2a) and the present day model (linear regression: *n* = 14, *r* ^2^ = 0.79, *p* < 0.0001; Fig. 2b). In both the historical and present-day data, short-distance migrants arrived earlier in the spring and took longer time to arrive at more northern sites, while long-distance migrants arrived later and were quicker to reach northern sites (Fig. 2).

**Fig. 3:**
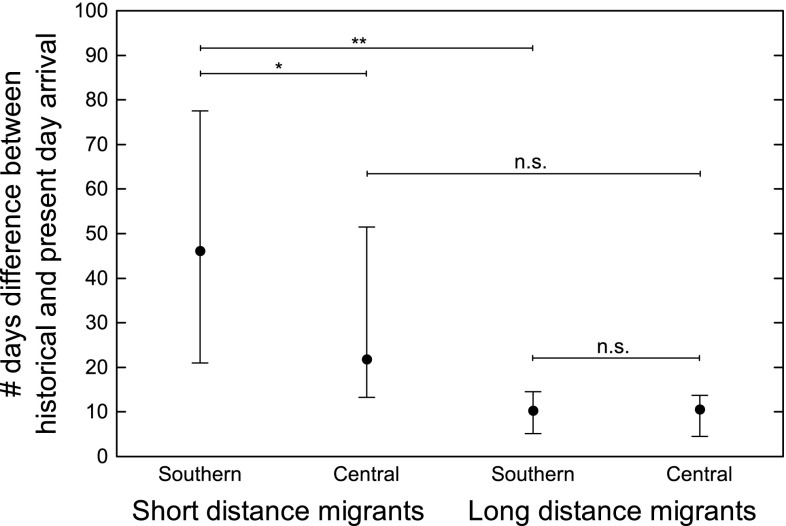
Difference in first arrival (median, 25 and 75% percentiles) between historical and present-day dataset in southern and central Sweden for short and long-distance migrants, respectively. Statistical *p* values from Mann–Whitney *U* test (comparing within latitude) and Wilcoxon matched pairs test (comparing within migration group) are indicated by *(*p* < 0.05), **(*p* < 0.01) and n.s. (*p* > 0.05)

To analyze changes over time in pattern of migration, we compared relative differences between historical and present-day datasets for short and long-distance migrants. Short-distance migrants had advanced their arrival to southern Sweden more over time than long-distance migrants (Mann–Whitney *U* test: *n*_short_ = 8; *n*_long_ = 6; *z* = 2.9; *p* < 0.01; Fig. 3). However, no such difference between long- and short-distance migrants could be seen in central Sweden (Mann–Whitney *U* test: *n*_short_ = 8; *n*_long_ = 6; *z* = 1.3; *p* = 0.2; Fig. 3). In long-distance migrants, the difference in first arrival between the historical and present-day dataset did not differ between southern and central Sweden (Wilcoxon matched pairs test: *n* = 6; *z* = 0.1; *p* = 0.9; Fig. 3). However, for short-distance migrants there was a larger difference in first arrival between the two data sets in southern than in central Sweden (Wilcoxon matched pairs test: *n* = 8; *z* = 2.2; *p* < 0.02; Fig. 3).

**Fig. 4:**
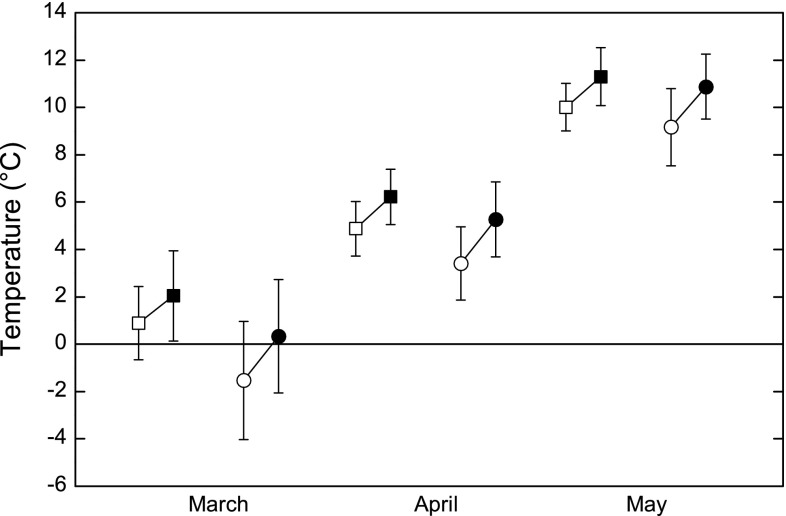
Mean (±SD) temperature for March, April, and May from the Swedish Meteorological and Hydrological Institute (SMHI) in southern (*squares*) and central Sweden (*circles*), respectively. Data are divided into historical data (1873–1917; *open symbols*) and present-day data (1984–2013; *filled symbols*)

Historical and present-day data on temperature for the six weather stations showed that spring temperature has increased between the historical and present day period. Due to low sample size, we could not analyze potential differences in the magnitude of temperature increase over time between southern and central Sweden, but in the six sampled sites average increase over time was larger in central than in southern Sweden for all spring months (south vs. central temperature increase: March: 1.3 vs. 1.8 °C, April: 1.4 vs. 1.9 °C and May: 1.4 vs. 1.7 °C (Table 2; Fig. 4).

**Table 2:** Historical and present day monthly mean temperature for the three sites in southern and central Sweden, respectively. Summary statistics are from Mann–Whitney *U* test comparing the historical and present-day data for each part of Sweden

Month	Southern Sweden					Central Sweden				
	Mean temperature (°C) ± SD		Difference in temp.	*p*	*z*	Mean temperature (°C) ± SD		Difference in temp.	*p*	*z*
	Historical data	Present-day data				Historical data	Present-day data			
March	0.8 ± 1.9	2.1 ± 2.1	1.3	<0.05	−2.5	−1.5 ± 2.5	0.3 ± 2.4	1.8	<0.01	−3.3
April	4.8 ± 1.3	6.2 ± 1.3	1.4	<0.001	−4.0	3.4 ± 1.5	5.3 ± 1.6	1.9	<0.001	−4.3
May	9.9 ± 1.2	11.3 ± 1.2	1.4	<0.001	−4.2	9.2 ± 1.6	10.9 ± 1.4	1.7	<0.001	−4.2

## Discussion

According to the calculated relationship between first day of arrival and latitude based on the historical and present-day datasets, all bird species arrived earlier today compared to the historical data, both in southern and central Sweden (Fig. 3). This is in line with observations from other studies comparing historical and present-day observations (e.g., Sparks 1999; Vitale and Schlesinger 2011). However, since our historical and present-day data were based on different sampling methods and analyses, we cannot draw any firm conclusion about to what extent the species have changed their arrival in time.

Data from six weather stations in southern and central Sweden show that spring mean temperatures have increased between the historical and present day period in both central (average 1.7 °C) and southern Sweden (average 1.4 °C). There are also indications for a larger temperature increase in central compared to southern Sweden. The potential difference is however small and is not reflected by corresponding latitudinal differences in changes of spring arrival in migratory birds.

Bird species arriving in early spring in southern Sweden (mainly short-distance migrants) take more time to colonize southern and central Sweden compared to species arriving later in spring (mainly long-distance migrants). This pattern is true for both the historical and present-day datasets (Fig. 2), and might be expected since bird species arriving late to southern Sweden have a shorter time span in which to accomplish their journey to the breeding grounds further north and to complete their reproductive phase. Observations of later arriving species progressing with higher speed from south to north have also been reported for British (Huin and Sparks 2000) and North American migrants (Hagan et al. 1991). Furthermore, long-distance migrants generally have a higher overall speed of migration compared to short-distance migrants (Alerstam and Lindström 1990; Hurlbert and Liang 2012). Interestingly, the relationship between first arrival date in southern Sweden and number of days to arrival in central Sweden is more or less the same for the two datasets. In other words, a bird species arriving to southern Sweden on the 1st of March will arrive around 25 days later in central Sweden, while a species arriving in southern Sweden on the 1st of May will arrive only a couple of days later in central Sweden, irrespectively if it happened a hundred years ago or today (Fig. 2).

A problem for this type of analyses is that the compared populations may over time change in the proportion of wintering and migratory birds. In our case, short-distance migrants have over time advanced their arrival to southern Sweden to a greater extent than long-distance migrants. This is at least partly due the fact that some individuals of the short-distance migrant species have included southern Sweden in their wintering area or shifted to resident behavior, giving misleading and very early estimates of the calculated migratory arrival dates at lower latitudes (Fig. 1b). According to annual first observation used in this study, this seems to be especially true for chaffinch, whooper swan, starling, and woodcock (Fig. 1b). It is well established that several short-distance migrants have become residents or shortened their migratory route during the last decades, possibly due to climate change enabling more northerly wintering areas (Newton 2008; Visser et al. 2009; for a review of the flexibility of migration systems, see Sutherland 1998). Among the species included in our study, the common crane is known to use more northerly wintering grounds (Alonso et al. 1991) and the common starling has in some populations shifted from migratory to partly resident behavior in central Europe (Berthold 1993).

The marked difference in arrival dates in southern Sweden between the historical and present-day data for the woodcock is worth reflecting over. We suggest that differences in sampling methods might explain the results. It is likely that, in the historical data, farmers documenting first arrival date to a larger extent recorded display flights (roding) of male woodcocks, while ornithologist today to a larger extent more specifically search for the first individual woodcocks by flushing birds in certain habitats. However, irrespective of this dissimilarity of sampling effort, there is a very large difference in first arrival between south and central Sweden in the present-day data, reflecting contemporary wintering behavior of some woodcocks in southern Sweden (Fig. 1b).

Both population size and sampling effort can affect reported date of first spring sight of a species and should thus be taken into consideration when comparing different time periods (Tryjanowski and Sparks 2001; Miller-Rushing et al. 2008a, c). It is however impossible to evaluate sampling effort and population sizes of the studied species for the historical period in our study. What we do know is that the whooper swan has increased in abundance during modern times thus possibly rendering an earlier arrival date than what would have been the case in the middle of the 20th century when the population size was much smaller than today (Svensson et al. 1999).

Difference in first arrival in central Sweden between historical and present-day datasets in short-distance migrants is not as large as for southern Sweden (Fig. 3). This shows that even though short-distance migrants arrive earlier to southern Sweden or use this area for wintering nowadays, it does not result in as early arrival in central Sweden. The difference in migratory change between southern and central Sweden suggests that an earlier spring onset influences northern latitudes less than southern latitudes, which is in accordance with what has been suggested in some earlier studies (Parmesan 2007; Rubolini et al. 2007). Consequently, we can conclude that the largest phenological change in this region will be seen in those species and areas where wintering starts to occur. So far, this effect has been strongest in the southern parts of the study area, which highlights the relevance of separating the effect of earlier arrival from a change in wintering areas.

For long-distance migrants, the difference in first arrival in historical and present-day datasets does not differ between southern and central Sweden, showing that there is less effect of climate change over latitude in long-distance migrants than in short-distance migrants. It is commonly argued that the migratory program of long-distance migrants is less responsive to climate change as it is rigidly, endogenously controlled, and governed by photoperiod (e.g., Berthold 1975; Gwinner and Wiltschko 1978; however, see Jonzén et al. 2006). Furthermore, due to unfavorable areas between breeding and wintering areas, long-distance migrants do not have the possibility to shorten the migration distance by gradually changing the wintering area (Pulido and Berthold 2010; but see Morganti and Pulido 2012). Short-distance migrants, on the other hand, are able to respond with more flexibility to the prevailing climate on their winter grounds which will correlate more with the conditions in Sweden, and also have the possibility to shift their wintering area northwards (Visser et al. 2009; Pulido and Berthold 2010), and for some individuals even to include southern Sweden as shown by this study.

In summary, our results confirm earlier findings that climate change has a larger effect on spring phenology in short-distance migrants than in long-distance migrants, and that the effect on short-distance migrants is larger in southern than in central Sweden. The results further suggest that the much earlier calculated arrival to southern Sweden among some short-distance migrants mirrors a change in wintering areas, hence, connecting migration phenology and wintering range shifts.
